# The Relationship Between Basic Psychological Needs Satisfaction and Career Adaptability Among University Students: The Roles of Grit and Career Decision-Making Self-Efficacy

**DOI:** 10.3390/bs15020167

**Published:** 2025-02-03

**Authors:** Min Xu, Haidong Lu, Jinlan Fu, Hairong Zhu, Yingfang Zhao

**Affiliations:** 1 School of Psychological and Educational Sciences, Zaozhuang University, Zaozhuang 277100, China; xumin@uzz.edu.cn (M.X.); fujinlan@uzz.edu.cn (J.F.); zhr@uzz.edu.cn (H.Z.); zhaoyingfang@uzz.edu.cn (Y.Z.); 2 School of Psychology, Northeast Normal University, Changchun 130024, China

**Keywords:** basic psychological needs satisfaction, career adaptability, grit, career decision-making self-efficacy, university students

## Abstract

Enhancing the career adaptability of university students is a practical necessity for addressing the challenge of student employment. This study explores the relationship between basic psychological needs satisfaction and career adaptability among university students based on Basic Psychological Need Theory and Social Cognitive Career Theory and constructs a corresponding chain mediation model. A survey was conducted among 635 university students from six provinces across China. The results indicate the following findings: (1) grit partially mediates the relationship between basic psychological needs satisfaction and career adaptability among university students; (2) career decision-making self-efficacy also partially mediates this relationship; and (3) grit and career decision-making self-efficacy serve as chain mediators in the relationship between basic psychological needs satisfaction and career adaptability. This study provides empirical support and significant guidance for enhancing the career adaptability development of university students.

## 1. Introduction

With the increasing prominence of the boundarylessness and dynamism in career development, unpredictability and change have become essential characteristics of the process (Bright & Pryor, 2005). In the face of the severe situation where the number of university graduates in 2025 is expected to reach 12.22 million, an increase of 0.43 million compared to the previous year, the employment trends of university students are also shifting from traditionally pursuing “iron rice bowls” to personalized and diversified career paths (Ma & Feng, 2021). Against this backdrop, the “14th Five-Year Plan for Employment Promotion” issued by the State Council clearly states the need to strengthen career education and employment and entrepreneurship guidance for university students to enhance their employability. However, internal surveys indicate that current university students exhibit significant deficiencies in career preparation and planning. Specifically, 72% of students perceive the employment situation as severe, while only 23% indicate that they understand their abilities and interests, and even fewer, at just 19%, have an in-depth understanding of the external professional environment. More seriously, up to 51% of students choose to abandon their goals after putting in effort for some time (M. Xu, 2017). This lack of career preparation makes it difficult for university students to transition from academic learning to workplace environments, leading to numerous challenges and difficulties in their employment process and highlighting the increasingly prominent employment issue (Wang et al., 2024b).

Career adaptability is a psychosocial capital for individuals to cope with tasks or environmental changes at different career development stages through self-adjustment (Savickas, 1997; Savickas & Porfeli, 2012). It is not only a core competency for university students and a core competency in university career guidance work (Ma & Feng, 2021) but also regarded as a critical factor in career development and career success (Savickas, 1997; Savickas & Porfeli, 2012; Lee, 2024). Therefore, exploring the influencing factors of career adaptability is a practical necessity to address the difficulty of student employment. From the perspective of Basic Psychological Need Theory, this study explores the relationship between basic psychological needs satisfaction and career adaptability, as well as the mechanisms of grit and career decision-making self-efficacy in this relationship, providing empirical evidence to enhance university students’ career adaptability, better cope with the challenges of career development and thereby promote their career success.

### 1.1. The Relationship Between Basic Psychological Needs Satisfaction and Career Adaptability

During transitions from school to work and from one job to another, individuals need to continuously adapt to environmental changes and face various adaptive challenges rather than established tasks (Savickas, 1997). Savickas defines career adaptability as a psychosocial resource or self-adjustment capacity for individuals to cope with current or anticipated career development tasks, career transitions, and career difficulties (Savickas & Porfeli, 2012; Lee, 2024; Hou et al., 2012). Career construction theory emphasizes that career development is essentially a dynamic constructive process of individuals pursuing the mutual adaptation between their subjective selves and the external world, with each individual’s constructive content and outcomes being different (Savickas, 1997). This theory posits that individual adaptivity facilitates the development of adaptability and adaptation and the process of adapting (Savickas & Porfeli, 2012; Savickas, 2020; Yu & Dong, 2019). Based on career construction theory, career adaptability is a type of adaptability (adaptive resource) that encompasses four dimensions: career concern, career control, career curiosity, and career confidence (Savickas, 2005). Career adaptability is regarded as an indispensable resource for individuals in the process of career development, pursuing career success, actively coping with various career challenges, and enhancing well-being (Johnston, 2018). Existing empirical evidence demonstrates that career adaptability plays a positive role in promoting individual career development. Specifically, career adaptability not only enhances academic performance and leads to higher academic achievements (Wang et al., 2024a) but also promotes an increase in life satisfaction (Santilli et al., 2017; Çarkıt, 2024), thereby facilitating the achievement of career success (Lee, 2024). Research on the influencing factors of career adaptability primarily focuses on individual factors. For example, optimism (Santilli et al., 2017), proactive personality (Wang et al., 2024b; Green et al., 2020), and psychological capital (Q. Yang et al., 2021; Q. Xu et al., 2024) have all been proven to facilitate the development of career adaptability. Among the limited studies on external environmental factors affecting career adaptability, peer support has a significant positive correlation with career adaptability and can positively predict its development (Agoes Salim et al., 2023). Social support factors such as teacher support, family support, and friend support have also been shown to have a positive impact on the development of career adaptability, with teacher support having a particularly prominent effect (T. Wang et al., 2023).

Basic Psychological Need Theory originates from Self-Determination Theory proposed by Deci and Ryan in the 1970s, which elaborates on the motivational process of how the external environment promotes internal motivation and the internalization of external motivation. Basic Psychological Need Theory suggests that humans are inherently motivated by three basic psychological needs: autonomy, competence, and relatedness (Deci & Ryan, 2000; J. Zhang et al., 2010). When the environment in which individuals are located satisfies these basic psychological needs, it effectively promotes the activation of internal motivation and the internalization of external motivation, thereby facilitating the development of individual behavior and mental health (J. Zhang et al., 2010). If any of these needs are unsatisfied or frustrated, they will have adverse effects on individual development (Wu et al., 2018). Researchers focusing on university students have demonstrated that the extent to which basic psychological needs are met can significantly contribute to enhanced levels of well-being (J. Yu et al., 2022). This finding concurs with research results obtained from adolescents across diverse cultural contexts (Chen et al., 2015). Nonetheless, despite the broad recognition of the impact of basic psychological needs satisfaction on individual well-being, research examining its effects on individual career development among university students remains comparatively sparse. In this context, researchers have commenced exploring the relationship between basic psychological needs satisfaction and individual career development. For example, studies targeting principals have identified a notable positive association between basic psychological needs satisfaction and job involvement (Toyama et al., 2022). Furthermore, meta-analytic investigations have revealed that basic psychological needs satisfaction exhibits a significant positive correlation with work effort and job satisfaction, while demonstrating a negative correlation with work−family conflict and turnover intentions (Van den Broeck et al., 2016). Research conducted on university students has indicated that basic psychological needs satisfaction can foster the development of career adaptability (M. Xu et al., 2023; Gulsen & Sahin, 2022). Concurrently, studies involving working professionals have shown that basic psychological needs satisfaction can promote career satisfaction (Yang et al., 2022a; Busque-Carrier et al., 2022).

**Hypothesis 1.**  *Basic psychological needs satisfaction can influence career adaptability.*

### 1.2. The Mediating Role of Grit

In recent years, the positive psychological traits of individuals, particularly grit, have garnered extensive attention from the academic community. Grit is defined as the perseverance and passion for long-term goals (Duckworth et al., 2007). Grit exerts a positive influence on individual psychological development. Existing research has confirmed that grit can enhance psychological security (Yang et al., 2022b) and effectively cope with interpersonal stress (Yang et al., 2022b), social exclusion, life disadjustment, and depressive states (Shin & Young, 2021), among other mental health issues. The role of grit in the realm of career cannot be overlooked as well. Studies have demonstrated that grit can promote career preparation behaviors (Byeong-Pyo & Park, 2021; Jongun & Hwa, 2021; Kim, 2021), elevate career maturity (Kwak et al., 2021; Ting & Datu, 2020), alleviate stress and obstacles in one’s career (Seo & Jongyeun, 2021) and thereby facilitate career success (Danner et al., 2020). As a crucial influencing factor of career adaptability (Çarkıt, 2024; M. Xu et al., 2023), grit has been shown to positively predict the development of career adaptability (Jung & Lee, 2021; So & Sujin, 2021; Dahye et al., 2021). This suggests that individuals with high levels of grit demonstrate stronger career adaptability when faced with challenges and changes in their career development (Wang et al., 2024b; Jung & Lee, 2021). Therefore, enhancing individuals’ grit levels is of significant importance in promoting the development of their career adaptability.

Furthermore, drawing from the theory of basic psychological needs, when individuals’ needs for autonomy, competence, and relatedness are met, their intrinsic motivation is strengthened, and their extrinsic motivation is internalized, thereby driving individuals towards positive and comprehensive development (Ryan, 2017). These three basic psychological needs serve as the core connecting the external environment with individual motivation and behavior. A review of the literature reveals only two relevant studies. Researchers have shown that there is a significant positive correlation between basic psychological needs satisfaction and grit among university students, and it can significantly positively predict the development of grit (W. Du et al., 2023; Marcelo-Torres et al., 2024). This implies that the satisfaction of individuals’ basic psychological needs contributes to the enhancement of their grit levels. When intrinsic motivation is strengthened, individuals’ enthusiasm and perseverance towards their goals will significantly increase, thereby promoting the development of their grit.

**Hypothesis 2.**  *Grit serves as a mediator between basic psychological needs satisfaction and career adaptability.*

### 1.3. The Relationship Between Basic Psychological Needs Satisfaction, Career Decision-Making Self-Efficacy, and Career Adaptability

Career decision-making self-efficacy (CDMSE) refers to an individual’s confidence in successfully completing relevant tasks during the career decision-making process (Betz & Hackett, 1981; Peng & Long, 2001). Career decision-making self-efficacy encompasses multiple aspects such as self-evaluation, information gathering, goal selection, planning, and problem-solving skills, exerting a profound impact on an individual’s career development (Taylor & Betz, 1983). Hackett and Lent further emphasize the importance of career decision-making self-efficacy in career planning, viewing it as an individual’s confidence in their problem-solving abilities, which helps unleash their potential and guides them towards pursuing careers of interest (Hackett et al., 1991). In career counseling practice, career decision-making self-efficacy is regarded as a key variable, showing a significant negative correlation with career decision-making difficulties (A. Zhou et al., 2024; S. Zhang et al., 2022). This implies that individuals with high career decision-making self-efficacy encounter fewer decision-making difficulties in the career decision-making process and can face career challenges with greater confidence.

Social Cognitive Career Theory (SCCT) emphasizes the interaction of individual, environmental, and cognitive factors in career development, with a particular focus on how individual behavior is influenced by cognitive factors (Lent et al., 1999). This theoretical framework provides a solid foundation for understanding the role of career decision-making self-efficacy in career development. As an important cognitive variable, career self-efficacy, compared to individual interests, values, and abilities, can more effectively predict career choice behavior and is a crucial factor in achieving career goals (Lent et al., 1994; Lent et al., 2003). Existing research has shown that there is a significant positive correlation between career decision-making self-efficacy and career adaptability and its various dimensions, with career decision-making self-efficacy significantly positively predicting individuals’ levels of career adaptability (S. Zhang et al., 2022; Li et al., 2021). Studies on different age groups, including high school students, adolescents (T. Wang et al., 2023), and university students (Agoes Salim et al., 2023; M. Xu et al., 2023), have all confirmed that career decision-making self-efficacy can predict the development of career adaptability.

Furthermore, the three basic needs have a significant impact on an individual’s career development. These basic needs serve as psychological nutrients, and all individual efforts are directed towards satisfying these innate needs. When these basic needs are met, they directly influence an individual’s growth, internalization, and well-being (Wu et al., 2018). Research has shown that the satisfaction of individuals’ basic psychological needs not only promotes the development of teaching self-efficacy but also enhances career satisfaction. In brief, the degree of satisfaction of basic psychological needs directly influences career satisfaction: satisfaction leads to increased satisfaction, while dissatisfaction leads to decreased satisfaction (Cai et al., 2018). Existing research has shown that basic psychological needs satisfaction among high school students can promote the development of self-efficacy in career decision-making (Cordeiro et al., 2015). This conclusion has also been verified among university students (M. Xu et al., 2023).

**Hypothesis 3.**  *CDMSE serves as a mediator between basic psychological needs satisfaction and career adaptability.*

### 1.4. The Chained Mediating Role of Grit and Career Decision-Making Self-Efficacy

In recent years, numerous studies have shown that grit significantly enhances the development of career decision-making self-efficacy. Research conducted among high school students (Byeong-Pyo & Park, 2021; Ting & Datu, 2020) and university students (Li et al., 2021) has revealed a strong positive correlation between grit and career decision-making self-efficacy. Grit positively predicts the advancement of career decision-making self-efficacy. Furthermore, studies focusing on nursing students (Kim, 2021) and dental students (Ji & Heo, 2021) have arrived at the same conclusion. This implies that individuals with high grit exhibit greater self-confidence when confronted with career decisions, believing in their capacity to triumphantly overcome various obstacles. This self-confidence motivates them to actively explore their inner selves and the external environment, engage more proactively in career planning and actively seek solutions, thereby enabling them to make more informed and beneficial career decisions for their personal development. Drawing from the above analysis and previously proposed research hypotheses, university students are at a crucial juncture transitioning from academic life to the workplace. During their career development, if their needs for autonomy, competence, and relatedness are fulfilled, they will possess stronger internal motivation, engage more actively in self-exploration and cultivate high levels of grit. This grit not only aids them in better adapting to career development and changes but also further promotes the development of career adaptability by bolstering their career decision-making self-efficacy.

**Hypothesis 4.**  *Grit and CDMSE have a chained mediating effect between basic psychological needs satisfaction and career adaptability.*

**Figure 1:**
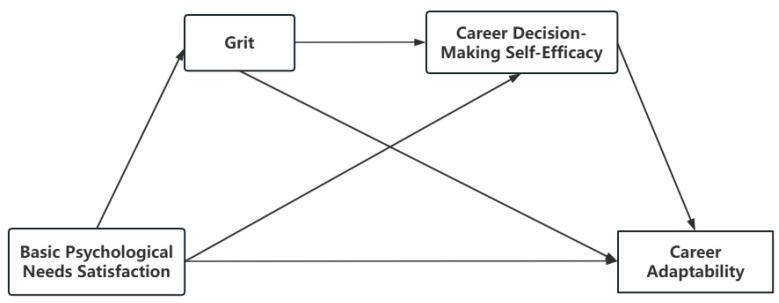
Hypothetical model.

In summary, this study explores the relationship between basic psychological needs satisfaction and career adaptability among university students, with grit and career decision-making self-efficacy playing multiple mediating roles (see Figure 1).

## 2. Materials and Methods

### 2.1. Participants

Convenience sampling was employed to select university students from universities across six provinces in China. The survey was conducted using the online platform Wenjuanxing and administered collectively to classes. A total of 703 questionnaires were collected. After rigorous screening and review, 69 invalid questionnaires were excluded, leaving 634 valid questionnaires with a response rate of 90.18%. Among the participants, there were 152 males (24%) and 482 females (76%); 419 participants (66%) hailed from rural areas, while 215 (34%) were from urban areas; 300 were first-year students (47.3%), 285 were second-year students (45%), 21 were third-year students (3.3%), and 28 were fourth-year students (4.4%); 258 participants (40.7%) majored in liberal arts and history, 219 (34.5%) majored in science and engineering, and 157 (24.8%) majored in the arts.

### 2.2. Instruments

#### 2.2.1. Basic Psychological Needs Satisfaction Scale

The Basic Psychological Needs Satisfaction Scale developed by Sheldon was adopted, which was translated and revised for university students by domestic scholars (Sheldon & Niemiec, 2006; J. Du et al., 2020). The scale consists of three dimensions with nine items, three items per dimension, covering autonomy, competence, and relatedness needs. A 7-point scoring system is used, with higher scores indicating a higher level of basic psychological needs satisfaction. In this study, the internal consistency reliability of the overall scale was 0.938.

#### 2.2.2. Grit Scale

The Grit Scale developed by Duckworth in 2007 (Duckworth et al., 2007) was adopted, specifically the Chinese version translated and revised by domestic scholars, known as the Grit-O Scale (Guan et al., 2015). The scale retains all 12 items and has an internal consistency reliability of 0.85. It consists of two dimensions with 12 items, 6 items per dimension, focusing on consistency of interests and perseverance of effort. A 5-point scoring system is used, with consistency of interests scored inversely and perseverance of effort scored positively. Higher scores represent a higher level of grit. In this study, the internal consistency reliability of the overall scale was 0.666.

#### 2.2.3. Career Decision-Making Self-Efficacy Scale

The Career Decision-Making Self-Efficacy Scale developed by Betz and Taylor, translated and revised for university students by domestic scholars was used (Peng & Long, 2001). The scale includes five dimensions with 39 items: self-appraisal (6 items), information gathering (9 items), goal selection (9 items), planning (8 items), and problem-solving (7 items). A 5-point scoring system is used, with higher scores indicating stronger self-efficacy in career decision-making. In this study, the internal consistency reliability of the overall scale was 0.978.

#### 2.2.4. Career Adaptability Scale

The Career Adaptability Scale developed by Savickas was adopted, with the Chinese version for university students revised by domestic scholar Hou Zhijin (Hou et al., 2012; Savickas & Porfeli, 2012). The scale consists of four dimensions with 24 items, 6 items per dimension, covering career concern, career control, career curiosity, and career confidence. A 5-point scoring system is used, with higher scores indicating stronger career adaptability. In this study, the internal consistency reliability of the overall scale was 0.955.

### 2.3. Research Procedure

This study was reviewed and approved by the Ethics Committee of the School of Psychology, Northeast Normal University (approval number: 2024021). To ensure the accuracy and validity of the data, the study adopted a centralized testing approach with classes as the unit. The researcher or corresponding teachers strictly followed the instructions, clearly explained the purpose and significance of the questionnaire to the participants and emphasized requirements such as confidentiality, the absence of right or wrong answers, and the need for independent responses. These measures were taken to ensure that each participant could correctly understand and successfully complete the questionnaire.

### 2.4. Data Processing

SPSS 22.0 was used to conduct common method bias testing and descriptive statistical analysis. The PROCESS plugin in SPSS 22.0 was employed to test the mediation effects.

## 3. Results

### 3.1. Analysis of Common Method Bias

Due to the potential issue of common method bias arising from self-report methods, the Harman’s single-factor test was employed to conduct exploratory factor analysis on all items (H. Zhou & Long, 2004). The results indicated that the variance explained by the first common factor was 30.45%, significantly below the critical threshold of 40%. Therefore, the data in this study were not affected by common method bias.

### 3.2. Descriptive Statistics and Correlation Analysis of Research Variables

Descriptive statistics indicate that the mean scores for university students’ basic psychological needs satisfaction (M = 44.50), grit (M = 36.96), career decision-making self-efficacy (M = 129.3), and career adaptability (M = 81.08) are all above the midpoint, suggesting that these constructs are at an above-average level among university students.

Correlation analysis reveals significant positive correlations among basic psychological needs satisfaction, grit, career decision-making self-efficacy, and career adaptability. Specifically, basic psychological needs satisfaction exhibits a weak positive correlation with grit and demonstrates significant moderate positive correlations with both career decision-making self-efficacy and career adaptability. Furthermore, grit is significantly and weakly positively correlated with career decision-making self-efficacy and significantly and moderately positively correlated with career adaptability. Lastly, there is a significant and strong positive correlation between career decision-making self-efficacy and career adaptability. Detailed results are presented in Table 1.

### 3.3. Mediation Effect Test

The mediation effect test was conducted using Model 6 in PROCESS, and the results are presented in Table 2. The results indicate that university students’ basic psychological needs satisfaction significantly and positively predicts grit, career decision-making self-efficacy, and career adaptability. Specifically, basic psychological needs satisfaction significantly and positively predicts grit (*β* = 0.230, 95% CI [0.101, 0.201]), career decision-making self-efficacy (*β* = 0.521, 95% CI [1.30, 1.657]), and career adaptability (*β* = 0.166, 95% CI [0.159, 0.353]) among university students. Grit significantly and positively predicts career decision-making self-efficacy (*β* = 0.237, 95% CI [0.752, 1.297]) and career adaptability (*β* = 0.206, 95% CI [0.353, 0.612]). Career decision-making self-efficacy significantly and positively predicts career adaptability (*β* = 0.542, 95% CI [0.259, 0.33]). Therefore, when both grit and career decision-making self-efficacy are included in the regression equation, basic psychological needs satisfaction still has a significant and positive predictive effect on career adaptability.

**Figure 2:**
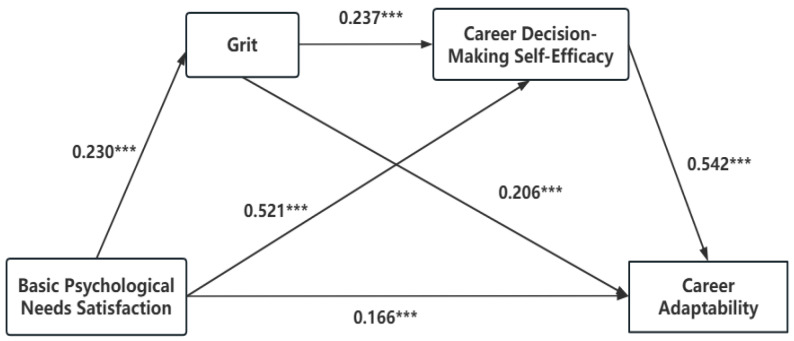
Diagram of the mediation model for grit and career decision-making self-efficacy (*** *p* < 0.01).

Using the Bootstrap procedure for significance testing of mediation effects, the confidence intervals corresponding to each path do not contain zero, indicating that all indirect effects are significant, as shown in Table 3 and Figure 2. They results indicate that grit and career decision-making self-efficacy mediate the relationship between basic psychological needs satisfaction and career adaptability. Specifically, the mediation effect with grit as the mediator accounts for 13.09% of the effect, the mediation effect with career decision-making self-efficacy as the mediator accounts for 78.55% of the effect, and the chained mediation effect with both grit and career decision-making self-efficacy accounts for 8.36% of the effect.

## 4. Discussion

Basic psychological needs satisfaction among university students can significantly and positively predict the development of career adaptability, confirming Research Hypothesis 1. This is consistent with previous research (M. Xu et al., 2023; Gulsen & Sahin, 2022), which indicate that not only is there a significant positive correlation between basic psychological needs satisfaction and career adaptability, but the satisfaction of these needs also significantly and positively predicts the development of career adaptability. In brief, when the basic psychological needs of university students are met, their level of career adaptability increases. The development of the theory of basic psychological needs further elucidates the underlying mechanisms of this relationship. As innate and intrinsic psychological factors, the three basic psychological needs positively promote the physical and mental health development of individuals (Deci & Ryan, 2000; J. Zhang et al., 2010). In university life, if students perceive a sense of control over their environment, establish good relationships with others and enjoy autonomy, these factors will enhance their sense of control and self-confidence in their careers, enabling them to face career challenges more bravely and adapt more flexibly to career changes, thereby promoting the development of their career adaptability. Conversely, if these three basic psychological needs are not met or are frustrated, the career development of university students, particularly the development of career adaptability, will be adversely affected. They may become passive and confused and lack self-confidence, finding it difficult to effectively cope with challenges and changes in their careers.

Grit partially mediates the relationship between basic psychological needs satisfaction and career adaptability, confirming Research Hypothesis 2. Basic psychological needs satisfaction has a significant and positive predictive effect on grit. This is consistent with previous research (W. Du et al., 2023; Marcelo-Torres et al., 2024). In brief, when the basic psychological needs of university students are met, their level of grit increases. These studies, along with the present one, explore the relationship between basic psychological needs satisfaction and grit. However, the present study further identifies grit as a partial mediator in the relationship between basic psychological needs satisfaction and career adaptability. Additionally, grit significantly predicts the development of career adaptability. This is consistent with previous research (Wang et al., 2024b; Jung & Lee, 2021). Career construction theory provides theoretical support for this finding (Deci & Ryan, 2000; J. Zhang et al., 2010), suggesting that grit can serve as an individual’s adaptability readiness, thereby promoting the development of career adaptability. When individuals possess high grit, they are better able to face challenges and difficulties in their career development, demonstrating stronger career adaptability. Therefore, when individuals’ basic psychological needs are met, their level of grit also increases. University students with high grit can maintain persistent interest and stable effort, maintain a positive mindset and action in the face of difficulties and challenges, continuously pursue their goals and interests and thereby promote the development of their career adaptability.

Research Hypothesis 3 is validated, as career decision-making self-efficacy plays a mediating role in the influence of basic psychological needs satisfaction on career adaptability. Basic psychological needs satisfaction has a significant positive predictive effect on career decision-making self-efficacy. This is consistent with previous research (M. Xu et al., 2023; Cordeiro et al., 2015). In brief, when university students experience sufficient autonomy, competence, and relatedness, they are more inclined to believe in their ability to make informed career decisions. Further research in this study shows that career decision-making self-efficacy significantly positively predicts career adaptability. This is consistent with previous research (Agoes Salim et al., 2023; M. Xu et al., 2023). University students with high levels of career decision-making self-efficacy demonstrate stronger adaptability when facing career changes and challenges. This aligns with Social Cognitive Career Theory, which focuses on how individuals’ career behaviors are influenced by cognitive factors, particularly career decision-making self-efficacy (Lent et al., 1994). Individuals with higher confidence in their ability to make career decisions are likely to be more confident and prepared when facing career challenges, thereby exhibiting stronger career adaptability. Therefore, when university students’ three basic needs are met, their career decision-making self-efficacy significantly increases, which in turn effectively promotes the development of career adaptability.

Grit and career decision-making self-efficacy play a chained mediating role in the relationship between basic psychological needs satisfaction and career adaptability, validating Research Hypothesis 4. Basic psychological needs satisfaction enhances university students’ grit, which indirectly enhances career adaptability by strengthening career decision-making self-efficacy. This finding further reveals the complex psychological mechanisms between basic psychological needs satisfaction and career adaptability. Grit significantly positively predicts career decision-making self-efficacy. This is consistent with previous research (Kim, 2021; Li et al., 2021). University students with a high level of grit tend to maintain a positive attitude and confidence when facing career decisions, believing in their ability to make the right choices. This self-confidence not only helps them remain rational when confronted with challenges but also motivates them to explore their environment, evaluate themselves and solve problems more actively, thereby enhancing their career adaptability. Furthermore, when university students’ basic psychological needs are met, they become clearer about their goals and maintain persistent effort and enthusiasm towards them. This clear goal orientation and sustained effort increase their confidence in making career decisions, convincing them of their ability to make wise choices. This boost in self-confidence further promotes the development of career adaptability, enabling university students to better cope with various challenges and changes in their career development. Therefore, in the future, by cultivating university students’ grit and enhancing their career decision-making self-efficacy, we can facilitate a comprehensive improvement in their career adaptability.

## 5. Conclusions and Educational Suggestions

This study has discovered that grit and career decision-making self-efficacy function as multiple mediators in the relationship between basic psychological needs satisfaction and career adaptability among university students.

This study provides empirical support and reference for enhancing the development of career adaptability among university students, offering significant guidance for helping them improve this skill. Firstly, attention should be paid to, and the basic psychological needs of university students should be met. Universities should focus on students’ basic psychological needs such as autonomy, competence, and belonging, providing them with more opportunities for autonomous choices and participation so that they can gain a sense of achievement and satisfaction through practice. At the same time, universities should establish positive teacher−student and peer relationships to enhance students’ sense of belonging and identity. Secondly, the cultivation of grit among university students should be emphasized. Universities can guide students to establish correct values and outlooks on life through group counseling activities, lectures, workshops, and other means, helping them clarify their future career goals of interest, develop a positive attitude towards difficulties and cultivate grit. Thirdly, the self-efficacy of career decision-making should be improved. Universities can carry out a series of diversified activities centered on career education, such as career planning education and employment guidance services, to help students understand their strengths and interests, clarify their career goals and development directions and improve their career decision-making abilities and self-confidence. Fourthly, the integration of mental health education and career education should be strengthened. By combining positive mental health education with career education, a mutually reinforcing virtuous circle can be formed. Mental health education can enhance university students’ positive psychological qualities, laying a solid foundation for career development. Meanwhile, career education can guide students to clarify their career goals and development directions, stimulating their intrinsic motivation and enthusiasm.

## 6. Limitations and Directions for Future Research

This study employed a convenience sampling method in terms of sample selection, which may have led to a lack of representativeness in the sample, thereby limiting the generality and applicability of the research findings. Future research could adopt random sampling or stratified sampling methods to ensure the representativeness and diversity of the sample. The current study employed a cross-sectional research approach to investigate the relationship between basic psychological needs satisfaction and career adaptability, as well as the underlying mechanisms. However, it is unable to determine the causal relationship between the variables. Future research could adopt a longitudinal research paradigm to further explore their interconnections. In terms of research methods, only quantitative research methods were used, without incorporating qualitative research. This may limit an in-depth understanding of the relationships between the variables. Future studies should employ a combination of quantitative and qualitative methods to gain a more comprehensive understanding of the relationship between basic psychological needs and career adaptability.

## Figures and Tables

**Table 1:** Descriptive statistics and correlation analysis of research variables.

Variables	*M*	*SD*	1	2	3	4
Basic psychological needs satisfaction	44.50	7.08	1			
Grit	36.96	4.65	0.228 ***	1		
Career decision-making self-efficacy	129.3	20.11	0.578 ***	0.356 ***	1	
Career adaptability	81.08	10.92	0.531 ***	0.439 ***	0.718 ***	1

*** *p* < 0.001.

**Table 2:** Regression analysis of the mediation model for grit and career decision-making self-efficacy.

Outcome Variables	Predictor Variables	*R*	*R* ^2^	*F*	*β*	Bootstrap 95% CI		*t*
						Lower Limit	Upper Limit	
Grit		0.248	0.061	8.217 ***				
	Gender				−0.021	−1.062	0.601	−0.544
	Hometown				0.015	−0.617	0.910	0.376
	Grade				−0.065	−0.878	0.068	−1.680
	Major				−0.074	−0.90	0.024	−1.860
	BPNS				0.230	0.101	0.201	5.390 ***
CDMSE		0.627	0.393	67.685 ***				
	Gender				0.004	−2.719	3.057	0.115
	Hometown				0.073	0.438	5.746	2.288 *
	Grade				0.017	−1.193	2.104	0.543
	Major				−0.008	−1.798	1.386	−0.254
	BPNS				0.521	1.30	1.657	16.233 ***
	Grit				0.237	0.752	1.297	7.039 ***
Career adaptability		0.759	0.576	121.41 ***				
	Gender				0.02	−0.814	1.812	0.746
	Hometown				0.054	0.041	2.464	2.03 **
	Grade				−0.022	−1.072	0.427	−0.846
	Major				0.001	−0.719	0.728	0.012
	BPNS				0.166	0.159	0.353	5.193 ***
	Grit				0.206	0.353	0.612	7.344 ***
	CDMSE				0.542	0.259	0.33	16.223 ***

CDMSE represents career decision-making self-efficacy; BPNS represents basic psychological needs satisfaction.* *p* < 0.05; ** *p* < 0.01; *** *p* < 0.001.

**Table 3:** Mediation effect analysis.

Effect	Pathways	Effect Value	Boot SE	Bootstrap 95% CI	
				Lower Limit	Upper Limit
Indirect effect 1	BPNS → Grit → Career adaptability	0.047	0.011	0.027	0.07
Indirect effect 2	BPNS → CDMSE → Career adaptability	0.282	0.025	0.237	0.331
Indirect effect 3	BPNS → Grit → CDMSE → Career adaptability	0.030	0.007	0.017	0.045
Total indirect effect		0.359	0.027	0.308	0.413

## Data Availability

The data presented in this study are available on request from the corresponding authors. The data are not publicly available due to containing information that may comprise the participants’ privacy.
